# Women’s experiences of personalised support for asthma care during pregnancy: A systematic review of the literature

**DOI:** 10.1186/s12884-017-1241-8

**Published:** 2017-02-20

**Authors:** Graham R. Williamson, Anita O’Connor, Elmslie-Jones Kayleigh

**Affiliations:** 1 School of Nursing and Midwifery, University of Plymouth, Drake Circus, Plymouth, Devon PL48AA UK; 2 Women’s Health Directorate, Royal Devon and Exeter Hospital NHS Trust, Barrack RD., Exeter, EX25DW UK

**Keywords:** Asthma, Pregnancy, Care, Support, Experiences

## Abstract

**Background:**

Asthma and pregnancy are both sources of anxiety for women. Although there has been a focus on physiological management of asthma and pregnancy, there has been little research on the impact that personalised support can have on asthma care during pregnancy. This systematic review and narrative synthesis of the literature set out to answer the question ‘What are women’s experiences of asthma care, its management and education, during pregnancy?’

**Methods:**

This systematic review was carried out using accepted methodology from the York Centre for Reviews and Dissemination. Electronic database searches were conducted using PsycInfo, CINAHL, MedLine, Google Scholar and the Cochrane Library, using the combination search terms: ‘Asthma’ AND ‘Pregnancy’ AND ‘Care’ AND (‘Education OR Information OR Experience’). Hand searching of journals and searches for grey literature were also undertaken. Independent quality appraisal by the three authors took place using the criteria detailed by Kmet et al. (Standard Quality Assessment Criteria for Evaluating Primary Research Papers from a Variety of Fields, 2004).

**Results:**

All papers scoring in excess of 60% were deemed to be of adequate quality for inclusion, of which there were five: two qualitative designs and three quantitative designs. The designs were too methodologically heterogeneous to permit statistical meta-analysis so narrative review and synthesis was undertaken. Despite an embryonic evidence bases, it is reasonable to conclude that personalised care has beneficial outcomes for pregnant asthmatic women.

**Conclusions:**

Larger randomised controlled trials investigating personalised care are required to build an evidence base which can establish the efficacy of such interventions.

## Background

Pregnancy can be a worrying time for women, and it is common that prospective mothers are anxious about the health of their child and about the birth itself; these worries are quite distinct from generalised anxiety and can manifest in prolonged mood disturbances which do not simply resolve after delivery [1]. In addition to these fears about pregnancy itself, pregnant women with asthma have an additional substantial health issue to worry about in their asthma. It is known that psychological morbidity including depression is in itself a predisposing factor for asthma exacerbations, and more specifically that anxiety is a cause of exacerbations in pregnant women [2]. This is compounded by the fact that during pregnancy, one third of pregnant asthma suffers’ asthma will worsen, one third’s will stay the same, and one third’s will improve and there seems to be little or no way of predicting accurately which individuals will be adversely affected [3].

Pregnant women with asthma are more likely to suffer pre-eclampsia than women without asthma, but if asthma is left uncontrolled, pregnancy complications can include hyperemesis, hypertension, pre-eclampsia, vaginal haemorrhage, complicated labour, fetal growth restriction, pre-term birth, increased perinatal mortality, neonatal hypoxia, and possibly increased Caesarean section risk [3]. United Kingdom guidance regarding the management of asthma and pregnancy [3] is that pregnant women with more than mild asthma should be monitored closely to make sure that their asthma is well controlled, which significantly reduces the risks of these pregnancy-related complications. As well as potential risks to their own health, pregnant women are likely to worry about the risks of their asthma and its treatments to their unborn baby, and to their new-born when breastfeeding. Guidance indicates that women should receive advice about maintaining good control of their asthma during pregnancy to avoid risks for mother and baby, and that B2 agonists, and oral and inhaled corticosteroids and other medications should be maintained where necessary during pregnancy [3]. European prescribing practice is reasonably consistent [4] with uniform peaks in prescribing for pregnant asthmatics in the second trimester (when exacerbations are likely to occur) and then a decline in the three months after birth. However, analysing the rates of prescribing and dispensing of medications says nothing about whether the women to whom they are prescribed understood what they were taking and why, or how worried they might have been when ingesting them.

Pregnant women are more likely to seek health advice and support for their asthma when they are pregnant than for many other aspects of pregnancy, particularly if they are of low income and in their first pregnancy [5]. Targeted health education and advice can have positive benefits for recipients in terms of confidence [6]. Self-management strategies can be beneficial [7, 8] but no single strategy is likely to suit all individuals [9]. Determining the extent to which such advice is delivered to pregnant asthmatic women is problematic. A recent systematic review of asthma care implementation interventions found that asthma self-management remains poorly implemented in clinical practice despite overwhelming evidence that asthma outcomes are improved [10], and despite guideline recommendations spanning thirty years. There is inconsistent knowledge and understanding of asthma and pregnancy amongst some midwives [11] who would routinely be in contact with pregnant women more often than other health professionals.

There is a wealth of research about managing physiological aspects of asthma and pregnancy including pharmacological interventions [12], and non-pharmacological interventions including education, progressive muscle relaxation and Fraction of Exhaled Nitric Oxide have also been researched, although the evidence base needs larger, high quality trials before results can be described as definitive [13]. However, there is a paucity of research examining pregnant asthmatic women’s experiences of their care, or the influence that healthcare professionals such as nurses and midwives can make, despite evidence that women’s asthma care in pregnancy is variable [14]. In order to explore these issues, we conducted this systematic review, the aim of which is to answer the question ‘What are women’s experiences of asthma care, its management and education, during pregnancy?’

## Methods

### Design

This systematic review followed the method set out by the Centre for Reviews and Dissemination [15], using specific search terms for database searches, inclusion and exclusion criteria and quality appraisal of relevant papers. In addition, we hand-searched references and also used Google Scholar to identify any other relevant papers. Types of study design to be included in the review were (see Table 1) empirical studies of any design, systematic reviews and framework/development studies. Exclusion criteria were: papers that were not published in English; not about education or informational care; were regarding children and their experiences or were experiences of healthcare professionals.

**Table 1:** Inclusion and Exclusion criteria applied to systematic review papers

Inclusion criteria	Exclusion criteria
Papers published between 2003 and 2016	Papers not published in English
	Children and their experiences
Adult females over 16 years of age with diagnosis of asthma	Experiences of healthcare professionals
	Not a systematic literature review/research study
Women who have undergone pregnancy	Not about education or informational care
	Letters/editorials/conference papers
	Low KMET score

### Search strategy

All searches took place in May 2016 and sought to identify peer reviewed articles published in English, between January 2003 and May 2016 (see Table 2 for the search results). A start date of 2003 was chosen as it was the date of publication of the first BTS/SIGN guideline. The electronic databases used were PsycInfo, CINAHL, MedLine and Google Scholar and the Cochrane Library, using the following combination of search terms: ‘Asthma’ AND ‘Pregnancy’ AND ‘Care’ AND (‘Education OR Information OR Experience’) with these last terms bracketed together as a concept.

**Table 2:** Number of papers identified through searching electronic databases

2003-2016 In English Search terms:	Medline	CINAHL (excluding Medline records)	PsycInfo	Cochrane Library	Google Scholar/Research Gate
Asthma + Pregnancy + Care + (Education OR Information OR Experience)	106	31	22	80	9

In an attempt to uncover grey literature, three further databases were searched using the key words ‘asthma and pregnancy’ and addressing the inclusion and exclusion criteria. Firstly, the PROSPERO database for systematic review protocols was searched but this search revealed no registered systematic review protocols on the topic. Secondly, a search of the clinical trials registry www.clinicaltrials.gov (a service provided by the US National Institutes of Health) revealed two relevant studies, however of these two, one was suspended and the second was completed in 2013 but showed no results posted in the register and a corresponding full article was not found during the more detailed search. Thirdly, the EU Clinical Trials Register (www. www.clinicaltrialsregister.eu) which contains registrations of trials to be conducted in the European Union (EU) or linked to the EU territories also revealed no relevant results. Therefore, the searches from PROSPERO, Clinicaltrials.gov and the EU Clinical Trials Register have not been included in the analysis.

**Fig. 1:**
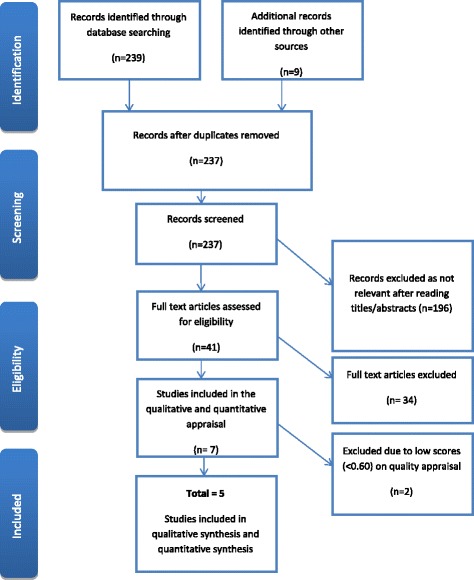
PRISMA

From the electronic databases that did produce results, 239 papers were identified with a further nine from additional sources. Figure 1 illustrates the process and is a Preferred Reporting Items for Systematic Reviews and Meta-Analyses (PRISMA) flow diagram. A total of 11 duplicates were then identified and removed, leaving 237 papers to be reviewed. After screening the titles/abstracts of these papers and applying the search inclusion and exclusion criteria to determine eligibility, a further 196 papers were excluded. The remaining 41 full articles were read in full, with an additional 34 being excluded as they did not meet the inclusion criteria or were not relevant.

### Quality appraisal

All papers were assessed using the quality appraisal tool for qualitative and quantitative research, as described by Kmet et al. [16]. Of the initial seven papers that were subjected to this quality appraisal, one was excluded due to lack of relevance [17] and one was excluded due to a low quality appraisal score and not being a research study [18], resulting in a total of five papers being included in the review. The three review authors assessed the papers independently and then compared and agreed scores. Where there were discrepancies in scoring, this was resolved through discussion until a consensus was reached.

## Results

The final papers included in the review consisted of two qualitative papers (see Table 3) that scored 90% [19] and 100% [14] respectively, and three quantitative papers (see Table 4) with scores ranging between 86 and 96%. The authors decided to include all papers scoring 60% or above, with only two papers in the initial assessment process not meeting this quality standard. All papers scoring in excess of 60% were deemed to be of adequate quality for inclusion within the review and a summary of these papers can be found in Table 5.

**Table 3:** Quality appraisal scores for Qualitative Studies

Papers	Chamberlain et al. 2014 [14]	Lim et al. 2012 [19]
Question/objective sufficiently described?	2	2
Study design evident and appropriate?	2	2
Context for the study clear?	2	2
Connection to a theoretical framework/wider body of knowledge?	2	2
Sampling strategy described, relevant and justified?	2	2
Data collection methods clearly described and systematic?	2	2
Data analysis clearly described and systematic?	2	2
Use of verification procedure(s) to establish credibility?	2	2
Conclusions supported by the results?	2	2
Reflexivity of the account?	2	0
Summary score	1.00	0.90

**Table 4:** Quality appraisal scores for Quantitative Studies

Papers	Grzeskowiak et al. 2016 [23]	Lim et al. 2014 [26]	Murphy et al. 2005 [22]
Question/objective sufficiently described?	2	2	2
Study design evident and appropriate?	2	2	2
Method of subject/comparison group selection or source of information/input variables described and appropriate?	1	2	1
Subject (and comparison group, if applicable) characteristics sufficiently described?	2	2	2
If interventional and random allocation was possible, was it described?	N/A	2	N/A
If interventional and blinding of investigators was possible, was it reported?	N/A	1	N/A
If interventional and blinding of subjects was possible, was it reported?	N/A	2	N/A
Outcome and (if applicable) exposure measure(s) well defined and robust to measurement/misclassification bias? Means of assessment reported?	2	2	2
Sample size appropriate?	2	2	2
Analytic methods described/justified and appropriate?	2	2	2
Some estimate of variance is reported for the main results?	2	2	2
Controlled for confounding?	0	2	1
Results reported in sufficient detail?	2	2	2
Conclusions supported by the results?	2	2	2
Summary score	0.86	0.96	0.91

**Table 5:** Summary of the studies

Reference and country	Aim	Study Design	Participants and sample	Findings
Chamberlain et al. (2014) [14] United Kingdom	To investigate in a qualitative study, the thoughts and feelings of women’s experiences of asthma in pregnancy.	Purposive sample. Semi-structured individual interviews were used to collect data. Interviews were transcribed and analysed using the ‘Framework’ Method.	*N* = 7 (22 women were asked to participate, however data saturation achieved at *n* = 7).	Themes: Asthma and pregnancy, Pregnancy and post-natal experiences and Health professionals.
Grzeskowiak et al. (2016) [23] Australia	To investigate the impact of introducing an antenatal asthma management service (AMS) on asthma control during pregnancy and subsequent perinatal outcomes.	Non-interventional prospective cohort study of pregnant asthmatic women attending a tertiary hospital antenatal clinic. A new nurse-led AMS was introduced offering asthma self-management education and support with outcomes.	Pregnant women (*n* = 169) recruited from antenatal clinic and followed up with visits at 12, 20, 28 and 36 weeks’ gestation by a midwife with additional respiratory training. *N* = 89 recruited prior to introduction of AMS and *n* = 80 recruited after AMS was introduced.	Relative risk for exacerbations, loss of control and persistent uncontrolled asthma were all reduced with attendance to AMS during pregnancy.
Lim et al. (2012) [19] Australia	To investigate how pregnant women manage their asthma during pregnancy and factors influencing their behaviour.	In-depth interviews (18 by telephone and 5 face-to-face) with pregnant asthmatic women. Framework Method.	A purposive sample (derived from 179 potential participants) of *n* = 23 asthmatic women at various stages of pregnancy and with varying severity of asthma using questions derived from the Asthma Control Questionnaire.	Themes: Risks versus Benefits, Self-Efficacy, Asthma as a Priority, Support and Guidance and Influences on Medication Use.
Lim et al. (2014) [26] Australia	To develop and evaluate a pharmacist-led intervention, directed at improving maternal asthma control, involving multidisciplinary care, education and regular monitoring to help reduce risks.	Randomized controlled trial. Participants were randomized to either an intervention or a usual care group. The primary outcome was Asthma Control Questionnaire (ACQ) score, with mean changes in ACQ scores compared between groups at 3 and 6 months to evaluate intervention efficacy.	*N* = 60 pregnant women <20 weeks’ gestation who had used asthma medications in the previous year were recruited.	Statistically significant difference between the two groups at 6 months (*p* < .001) with all participants in the intervention group having ACQ scores of <1.5 indicating adequately controlled asthma.
Murphy et al. (2005) [22] Australia	To determine the level of asthma self-management skills and knowledge among pregnant subjects and describe the implementation of an asthma education programme delivered in an antenatal clinic setting.	Pregnant subjects with asthma were assessed by an asthma educator at 20 and 33 weeks’ gestation. Some were provided with a written action plan.	Pregnant subjects with a doctor diagnosis of asthma (*n* = 211) were recruited. *N* = 149 females were followed up at ~33 weeks’ gestation. All were seen by the same asthma educator.	Significant improvements found in all aspects of asthma self-management.

Certain methodological features usually associated with systematic reviews have not been possible to assess in this study [20]. Assessment of heterogeneity in the results of systematic reviews is important because it indicates whether in fact the studies to be included in the review are similar enough for that combination to be meaningful. Inevitably, study variables, units of measurement and outcomes will be dissimilar, but the extent of that difference should be assessed statistically where possible when meta-analysis using odds ratios is intended [21]. The results of this systematic review are methodologically diverse, meaning that they are too heterogeneous in study designs for their outcomes to be combined in statistical meta-analysis and the papers are therefore evaluated and discussed below in a narrative review, beginning with those from qualitative designs and moving on to the quantitative designs. This heterogeneity, combined with a near-absence of randomised controlled trials, means that it has not been possible to estimate publication bias or produce funnel plots to analyse it; however, we have no reason to believe that publication bias is a significant issue in this review or indeed that it would alter the analysis and synthesis of studies, because publication bias is most important if it influences the interpretation and direction of effect sizes in meta-analysis, which this systematic review is not able to do [20].

## Discussion

### Qualitative designs

Lim et al. [19] and Chamberlain et al. [14] conducted interview-based qualitative research with purposive samples of women (23 and seven participants respectively). Chamberlain et al. [14] interviewed women who had delivered their babies within two years of the interview, using ‘Framework Analysis’ to construct themes from the qualitative data they recorded. Key findings were themes related to women’s experiences of Asthma and pregnancy, which were largely concerning their experiences of illness and related physical health anxieties; Pregnancy and post-natal experiences, where they related concerns about their asthma and its potential impact on their baby; and Health professionals, in which the women discussed mostly negative experiences with health professionals. The major limitations of this study [14] concern the extent of women’s symptoms, in that they did not objectively measure asthma symptoms using a valid and reliable scale such as the Asthma Control Questionnaire (ACQ), but instead relied on ‘a confirmed diagnosis of asthma’ as their diagnostic categorisation. A further limitation concerns the interpretation of findings, in that participants in Chamberlain et al’s study [14] may have had an element of recall based on the length of time since delivery (which is not reported for participants), although it is not clear if that might lead them to over-or under-represent their experiences.

Lim et al. [19] recruited a purposive sample from one Australian hospital (derived from 179 potential participants) of *n* = 23 asthmatic women who were still pregnant, either primi- or multi-gravid, as well as newly delivered within five weeks, and sought to investigate how pregnant women manage their asthma during pregnancy. They conducted in-depth interviews (18 by telephone and 5 face-to-face) with pregnant asthmatic women, which were again coded using the ‘Framework approach’. Five major themes relating to asthma management during pregnancy and changes in behaviour emerged: Risks versus Benefits, Self-Efficacy, Asthma as a Priority, Support and Guidance and Influences on Medication Use. Asthma monitoring during pregnancy was seen as a low priority for some women and their doctors. Communication between pregnant women and health professionals regarding asthma management was poor.

Arguably Lim et al. [19] were not comparing the perspectives of a similar enough sample by recruiting from across the course of pregnancy and delivery, however they were accurate in their assessment of symptoms, using the ACQ to assess their participants’ symptoms. The largest potential methodological weakness of Lim et al. [19] is the lack of explicit discussion of rigour and reflexivity in their account, although this is a comparatively minor point of critical analysis in an otherwise robust qualitative design.

Despite these differences in recruitment and classification, both qualitative studies produced similar themes and, in some areas, overlapping findings. It is striking that participants in both studies talked about their fears explicitly and this centred on medications, particularly steroids. In both studies, the women reported worry about risks versus benefits to their health and that of their babies of taking their medications, with some women in Lim et al’s study [19] unilaterally reducing their medications without reference to health care professionals. Participants in both studies talked about their desire for self-management, but most were unaware that their asthma might worsen in pregnancy and were unprepared for that. Most wanted ‘more’ education with little focus on community sector health care personnel including midwives and general practitioners. Lim et al’s study [19] indicated that women used the internet for information, but this was not discussed by Chamberlain et al’s [14] participants. As Lim et al. [19] was conducted in Australia and Chamberlain et al. [14] was conducted in England, these two papers indicate that these issues are not restricted to one location.

### Quantitative designs

Two of the quantitative papers [22, 23] were quasi-experimental designs with pre-and post-tests applied to non-randomised groups of pregnant asthmatic women.

Murphy et al. [22] recruited 211 participants with diagnosed asthma who were also pregnant, who were then assessed by a specialist nurse at 20 weeks’ gestation and then followed up at 33 weeks’ gestation by the same nurse. As part of these assessments, an information session consisting of history taking, self-management skills, medications adherence and knowledge, and further education was given to participants, contributing to a written action plan. Peak Flow (PEF) self-measurement and Forced Expiratory Volume at one second (FEV1) were recorded and urgent medical review was secured where necessary. Murphy et al. [22] report that at first visit, the women had poor self-management skills, 40% non-adherence to inhaled corticosteroids and little understanding of medications. Only 3% were taking their own PEF. After the session with the specialist nurse, all of these metrics were substantially improved with women who remained in the study. Specifically, those who received a written asthma action plan (WAAP) had babies with statistically significantly higher birth weight than those who did not.

The major strength of a quasi-experimental study design with pre-and post-tests lies in its pragmatism as it explores important concepts in real-world situations. Limitations in Murphy et al’s [22] study design include that pre-post-test designs do not eliminate Hawthorne effects and are potentially open to investigator bias via recruitment and selection. Also, there is likely to be a significant maturation effect (where test scores improve as a result of time passing and participants developing in some way) which is a potential source of threat to internal validity, casting doubt over the credibility of such study designs as evidence for efficacy of treatment effects [24, 25]. and despite the issues with Hawthorne effects, potential sources of bias and threats to internal validity [24, 25] noted above, it is clear that their intervention, involving contact and care from a specialist nurse, made clinically important differences to their women and babies, and that this was particularly the case for those most severely asthmatic during their pregnancy.

In a similar design to that of Murphy et al. [22], Grezeskowiak et al. [23] recruited 169 pregnant women with diagnosed asthma. These women were assessed at four points during their pregnancy by a midwife with additional respiratory training for demographic variables including socio-economic status as well as asthma-related ones including medications, control of symptoms (using the Asthma Control Questionnaire, ACQ), use of corticosteroids and hospital admissions. FEV1 was recorded. Half way through the study a new antenatal asthma service was introduced which was led by a respiratory nurse, meaning that the women received sessions on managing asthma in pregnancy based on evidence-based guidelines. Outcomes were assessed for the 89 women who received standard care and to 80 who attended the new service. Similar to Murphy et al’s study [23], Grezeskowiak et al. [23] found that those who attended the new antenatal asthma and pregnancy service, involving contact and care from a specialist nurse, were statistically significantly less likely to have a loss of asthma control or persistent symptoms during their pregnancy. There was an increase in smoking cessation amongst those in the intervention group compared to the control group, as the asthma management nurses were trained to provide advice on how to stop smoking. Perinatal outcomes were the same but there was a non-statistically significant reduction in pre-term births and Caesarean sections in those attending the new service. Indeed, several variables were non-statistically significant; for example, there was no difference in the reduction of exacerbations in moderate/severe asthma between the two groups, indicating that, as well as the criticisms of quasi-experimental pre-post-tests designs discussed above [24, 25], Grezeskowiak et al’s [23] study was underpowered and the authors acknowledge this. Study power is important because it is difficult to assess whether the non-significant results are genuine or are type II errors (false negatives) resulting from small sample size [25], which is a potential threat to the external validity of this study.

Despite the limitations noted in both the quasi-experimental designs [22, 23] Grezeskowiak et al’s [23] results support those of Murphy et al. [22], indicating that targeted intervention involving contact and care from a specialist nurse can make a difference in clinical outcomes. Grezeskowiak et al. [23] speculate that the asthma management nurses provided a continuity of care which is particularly valuable for women at the lower end of the socio-economic scale, as these women are generally more likely to fare badly in many pregnancy outcomes including vaginal delivery, pre-term delivery and miscarriage.

Lim et al. [26] conducted the only randomised controlled trial retrieved in our systematic review, which included pregnant women up to 20 weeks’ gestation who had asthma symptoms within the last 12 months. Participants were assessed for severity of symptoms and then randomised into mild to intermittent or moderate to severe asthma symptom groups, and within each group block randomisation was applied to allocate participants to either usual care (control) group or multidisciplinary care (intervention) group. The intervention was a Multidisciplinary Approach to the Management of Maternal Asthma (MAMMA©) and included education, monitoring, feedback and follow-up on a monthly basis, including assessment using FEV1 and the ACQ. The intervention consisted of regular contact, advice and medication review by a pharmacist, who referred women with worsening asthma to a family physician for further review and support. Comparison of the ACQ scores at 3 and 6 months were the primary variables, and secondary variables were asthma exacerbations, hospital admissions, time off work, oral steroid use and neonatal outcomes (the latter obtained from obstetric notes). An 80% power to detect a ACQ score of >0.5 would be achieved with 29 participants. Lim et al. [26] recruited 60 in total and found that 70% of participants did not realise the importance of good asthma control at the start of the trial and 19 had reduced or stopped their medications because they had become pregnant. In the intervention group, the ACQ score reduced at 3 and 6 months, although it was only statistically significant at 6 months, and all the scores in the intervention group indicated adequate asthma control. Perinatal outcomes were the same for intervention and control groups.

Their trial was adequately powered, and Lim et al. [26] applied intention to treat analysis (ITT) for drop outs, meaning that missing patients’ data was not omitted from the study, which might otherwise undermine the theoretical basis for statistical analysis and is a source of bias towards favourable outcomes [27, 28]. As a single-blinded study, one potential criticism is that the Hawthorne effect may bias outcomes, and this has been shown to be a particularly significant issue in studies where participants in the intervention group receive additional follow up compared to those in the control group [28]. However, in order to conduct research of this nature in the ‘real world’ a pragmatic approach is required [29], and so it would not have been possible for Lim et al. [26] to double blind participants concerning their group allocation. Furthermore, data collection and analysis were undertaken by blinded independent researchers, meaning that group allocation was concealed to avoid bias.

### Synthesis, hierarchy of evidence and clinical effectiveness

It is clear from the results of this systematic review and narrative summary that a very important factor in securing beneficial outcomes for pregnant asthmatic women is targeted support from a dedicated healthcare professional who is able to help women with management and self-management. Personalised contact featured in all five of the studies reported here: in the two qualitative designs [14, 19] it was described as important but lacking, whilst the three quantitative designs [22, 23, 26] gave positive evidence concerning the beneficial impact that could be achieved with targeted input and personal contact. These quantitative designs [22, 23, 26] are particularly important in this regard as they have quantified a range of clinically and statistically important benefits accruing from dedicated asthma care including improvements in asthma symptoms measured by ACQ scores, pre-term births, birth weight and Caesarean section rates.

However, synthesising and interpreting the results of this systematic review is highly tentative. The purpose of a hierarchy of evidence is to evaluate the strength of evidence in relation to clinical effectiveness [30]. Accepted hierarchies are diagrammatically pyramidal and graduated with systematic reviews and meta-analyses as the most effective designs, randomised controlled trials (RCTs) second, with other quantitative designs and then qualitative designs as progressively less effective guides for clinical decision making. The five papers reviewed in this study are methodologically heterogeneous. Lim et al. [26] is an RCT, and so sits highest in traditional hierarchies of evidence, but even so it is single blinded only and a weaker design to RCTs that are double blinded and can more fully control potential biases [30]. The before and after studies [22, 23] are weaker designs still, with concerns about internal validity accruing from potential Hawthorne and maturation effects as well as investigator biases [24]. Lim et al. [19] and Chamberlain et al. [14] are qualitative designs that, although robust in that paradigm, are likely to continue to be regarded as poor evidence for clinical effectiveness despite efforts to explore and delineate qualitative designs as evidence for practice [31].

In summary, the evidence base concerning personalised care for pregnant asthmatic women can best be described as embryonic and further research is required to discover the best strategies for helping them to self-manage their asthma. This makes it difficult to offer firm recommendations for clinical practice as a result of this systematic review and narrative synthesis, however, it seems reasonable to suggest that personalised care and contact with healthcare professionals can have beneficial outcomes, and this has been established in community settings [32, 33]. This recommendation would rank as ‘weak’ according to GRADE classification [34] but our ‘weak’ findings complement the ‘strong’ recommendations made in clinical guidelines that women should receive advice about the importance of maintaining good asthma control during pregnancy to avoid problems for mother and baby, and that pregnant women with moderate/severe asthma should be closely monitored to keep their asthma well controlled [3].

### Recommendations for future research

It seems clear from this systematic review that pregnant women with asthma need additional support and care relating to their asthma. The papers reviewed here indicate that contact with a dedicated healthcare professional can facilitate that, and further research is required to examine this area, with larger trials that establish whether or not there is any ‘added value’ in personalised support for pregnant women with asthma, over and above any gains that might be made from different therapeutic regimes and/or asthma control monitoring using Fraction of Exhaled Nitric Oxide, particular as this latter monitoring option is not necessarily diagnostic [3].

Authoritative guidelines recommend this level of detailed and personalised response from health care professionals [3], but if resources or practitioner confidence do not permit such close intervention in the community [35] telehealth solutions might provide a method to combine asynchronous information access and improve self-efficacy [36]. Although the role of telehealth in asthma has been evaluated in a Cochrane systematic review and found not to result in clinically important improvements in asthma quality of life in mildly asthmatic people, telehealth interventions are useful in those with severe disease at high risk of hospital admission [36], and also to be effective in reducing anxiety and improving quality of life in patients with Chronic Obstructive Pulmonary Disease [37]. Furthermore, when specifically targeted at pregnant asthmatic women, telehealth has proved much more successful: where participants were offered personal written asthma action plans, a smart-phone application (app) and a hand-held spirometer, there were statistically significant improvements in asthma quality of life as measured using the ACQ [38]. This type of service delivery has the advantage of involving participants in self-monitoring and disease regulation, particularly when made gender-specific and acknowledging hormonal variations [39] as would be the case for pregnant women. Pregnant asthmatic women already use the internet for information [40] and it is therefore reasonable to assume that a structured approach would be beneficial, but this requires appropriate scientific analysis. One recommendation for further research, therefore, is to examine the role of telehealth in provision of information to pregnant asthmatic women. More specifically, there could be benefits in the provision of resources that are available asynchronously for personal consultation by women at times convenient to them, as an adjunct to WAAPs, rather than relying on timetabled contact during ‘office hours’ with dedicated professionals. One systematic review [40] found consistent evidence suggesting that asynchronous telehealth lead to shorter waiting times, fewer unnecessary referrals, high patient satisfaction, and no lesser diagnostic accuracy when compared with face-to-face consultations. This asynchronicity has been found to be particularly important in paediatric asthma, where emergency admissions were avoided and satisfaction with the resources was high [41] and substantial costs savings in remote locations [42]. Such asynchronous resources could act as an information repository to provide reassurance and guidance about important aspects of asthma care including symptom control and medications, and might help pregnant women to be more confident about when to contact health professionals and then to get more out of their consultations with them. This might be particularly useful as women in pregnancy are uniquely receptive to educational input, already access the internet for information [40]. Clearly the internet offers a means for social support which is accessible from within the home 24 h a day, and has already been shown to be effective in supporting pregnancy, including in reducing anxiety [43].

## Conclusions

We found an embryonic evidence base concerning the benefits of personalised support, with only five papers that meet our inclusion and exclusion criteria and quality assessment scoring [16]. Whilst the standing of this evidence base is low in relation to accepted hierarchies of evidence, and the strength of our recommendations consequently weak [34], they support the authoritative guidance [3] that pregnant asthmatic women should receive monitoring and advice which helps them to understand their asthma symptoms and the role that their medications play in controlling them, including inhaled B2 agonists, and oral and inhaled corticosteroids. Larger randomised controlled trials investigating personalised care are required to build an evidence base to establish the efficacy of such interventions.

## Abbreviations

ACQ: Asthma Control Questionnaire
FEV1: Forced expiratory volume at one second
GRADE: Grading of Recommendations Assessment, Development and Evaluation
MAMMA©: Multidisciplinary Approach to the Management of Maternal Asthma
PEF: Peak expiratory flow
PRISMA: Preferred Reporting Items for Systematic Reviews and Meta-Analyses
RCT: Randomised controlled trial
WAAP: Written asthma action plan
